# Serum glial fibrillary acid protein associates with TSPO-expressing lesions in multiple sclerosis brain

**DOI:** 10.1177/17562864251352998

**Published:** 2025-07-28

**Authors:** Tanja Sjöros, Maija Saraste, Markus Matilainen, Marjo Nylund, Mikko Koivumäki, Jens Kuhle, David Leppert, Laura Airas

**Affiliations:** Turku PET Centre, University of Turku, Åbo Akademi University, Turku University Hospital, P.O. Box 52, Turku 20521, Finland; Clinical Neurosciences, University of Turku, Turku, Finland InFLAMES Research Flagship, University of Turku, Turku, Finland; Turku PET Centre, University of Turku, Åbo Akademi University, Turku University Hospital, Turku, Finland; Clinical Neurosciences, University of Turku, Turku, Finland; InFLAMES Research Flagship, University of Turku, Turku, Finland; Neurocenter, Turku University Hospital, Turku, Finland; Turku PET Centre, University of Turku, Åbo Akademi University, Turku University Hospital, Turku, Finland; Clinical Neurosciences, University of Turku, Turku, Finland; InFLAMES Research Flagship, University of Turku, Turku, Finland; Turku PET Centre, University of Turku, Åbo Akademi University, Turku University Hospital, Turku, Finland; Clinical Neurosciences, University of Turku, Turku, Finland; InFLAMES Research Flagship, University of Turku, Turku, Finland; Neurocenter, Turku University Hospital, Turku, Finland; Turku PET Centre, University of Turku, Åbo Akademi University, Turku University Hospital, Turku, Finland; Department of Neurology, University Hospital and University of Basel, Basel, Switzerland; Departments of Biomedicine and Clinical Research, Multiple Sclerosis Centre and Research Center for Clinical Neuroimmunology and Neuroscience (RC2NB), University Hospital and University of Basel, Basel, Switzerland; Department of Neurology, University Hospital and University of Basel, Basel, Switzerland; Departments of Biomedicine and Clinical Research, Multiple Sclerosis Centre and Research Center for Clinical Neuroimmunology and Neuroscience (RC2NB), University Hospital and University of Basel, Basel, Switzerland; Turku PET Centre, University of Turku, Åbo Akademi University, Turku University Hospital, Turku, Finland; Clinical Neurosciences, University of Turku, Turku, Finland; InFLAMES Research Flagship, University of Turku, Turku, Finland; Neurocenter, Turku University Hospital, Turku, Finland

**Keywords:** 18 kDa translocator protein, astrocyte, brain imaging, glial fibrillary acid protein, magnetic resonance imaging, microglia, multiple sclerosis, neuroinflammation, positron emission tomography

## Abstract

**Background:**

Serum glial fibrillary acidic protein (sGFAP) is a promising biomarker for multiple sclerosis (MS) disease progression. Elevated sGFAP levels are considered to reflect ongoing astrocyte-related pathology in the central nervous system.

**Objectives:**

To study whether sGFAP levels associate with 18 kDa translocator protein (TSPO) availability in MS brain. TSPO is a mitochondrial molecule that is expressed by activated microglia and astrocytes.

**Design:**

Cross-sectional multimodal biomarker correlation study.

**Methods:**

We included 80 people with MS (66 relapsing-remitting and 14 progressive MS, 69% women), and 11 healthy control participants (73% women). sGFAP was measured using single molecule array (Simoa®) technology in combination with 3T magnetic resonance imaging and positron emission tomography (PET) using a TSPO-binding [^11^C]PK11195 radioligand.

**Results:**

sGFAP was higher among people with progressive MS (median 122 pg/ml) compared to healthy controls (median 59 pg/ml, *p* = 0.0002) or participants with relapsing-remitting MS (median 77 pg/ml, *p* = 0.0056). Among people with MS, higher sGFAP associated with higher volume of chronic lesions with increased TSPO activity (*r* = 0.36, *p* = 0.0011) and with thalamic TSPO activity (*r* = 0.30, *p* = 0.0069), as well as with T1 and T2 lesion loads (*r* = 0.38, 0.41, *p* = 0.0005, 0.0002, respectively). Smaller normal-appearing white matter (*r* = −0.36, *p* = 0.0009), cortical gray matter, and thalamus volumes (*r* = −0.39, *p* = 0.0003 for both) correlated with higher sGFAP. In regression analyses, the volume of TSPO-expressing lesions, together with age and MS disease-modifying treatment status, explained 27% of the variation in sGFAP.

**Conclusion:**

sGFAP associates with adverse magnetic resonance imaging and PET imaging outcomes. The association between a high prevalence of TSPO-expressing white matter lesions and high sGFAP suggests that lesion-associated glial activity promotes MS progression partially via astrocyte-driven mechanisms. A combination of various soluble biomarkers and PET ligands for specific cell types may add to the understanding of progression-promoting cellular mechanisms in the brain.

**Trial registration:**

ClinicalTrials.gov NCT03134716, NCT03368677, NCT04126772, NCT04239820, https://clinicaltrials.gov.

## Introduction

Glial fibrillary acidic protein (GFAP) is the main intermediate filament of astrocytes. The level of GFAP in blood is considered to reflect the quantity of reactive astrocytosis in the central nervous system (CNS).^1^ Serum soluble glial fibrillary acidic protein (sGFAP) is considered a promising biomarker for multiple sclerosis (MS) disease progression.^2,3^

Generally, MS pathogenesis contains both adaptive and innate immune system activation processes. Particularly, the adaptive immune system is associated with relapses and formation of focal inflammatory lesions in the CNS, whereas the innate immune system contributes to CNS-contained diffuse inflammation and neurodegeneration that clinically manifests as disease progression independent of relapse activity (PIRA).^4^ In addition, astrocytes are among the key players in MS pathology and lesion development.^5–8^

Previously, we have reported that higher concentrations of sGFAP are associated with higher Expanded Disability Status Scale (EDSS) score, older age, longer disease duration, progressive disease course, and magnetic resonance imaging (MRI) pathology among patients with relapsing-remitting MS (RRMS) or progressive MS.^9^ In addition, high sGFAP is associated with myelin and axonal loss in the normal-appearing white matter (NAWM) measured with diffusion tensor imaging (DTI), supporting the concept of sGFAP as a marker of progression-associated MS pathology.^10^ Baseline sGFAP has predicted later PIRA and future need for gait aid.^11,12^ In addition, sGFAP remained elevated during a five-year follow-up among patients with PIRA.^11^ Therefore, sGFAP is a plausible candidate for a soluble marker of smoldering disease activity.

Positron emission tomography (PET) using the radioligand [^11^C]PK11195, which binds to the 18 kDa mitochondrial translocator protein (TSPO), is a method to assess the activation of microglia, macrophages, and astrocytes.^13^ Moreover, TSPO expression has also been observed in a subset of endothelial cells.^13^ Microglia and astrocytes are frequently co-activated, and crosstalk between activated microglia and neurotoxic reactive astrocytes takes place in neurodegenerative diseases and contributes to their progression.^14–16^ TSPO-PET imaging has been used to verify that glial activation associates with the risk of later MS progression,^17–21^ and it can also be applied to address longitudinal changes in glial activation in MS brain.^22,23^

The aim of the current study was to evaluate whether sGFAP associates with increased TSPO-availability among patients with RRMS and progressive MS. To our knowledge, this is the first study assessing the associations between sGFAP and TSPO-PET measurable glial activation in MS lesions and in other brain areas of interest, including the thalamus.

## Methods

The study was conducted at the Turku PET Centre, University of Turku, and Turku University Hospital, Turku, Finland. The study was conducted according to the Declaration of Helsinki, approved by the Ethics Committee of the Hospital District of Southwest Finland, and reported according to STROBE guidelines for cross-sectional studies.^24^

### Participants

Study patients were recruited between 2016 and 2022 from the outpatient clinic of the Division of Clinical Neurosciences at the Turku University Hospital, Turku, Finland, based on MS diagnosis according to the criteria at the time of the diagnosis and willingness to participate in a PET study. Five participants had their original diagnosis based on the Poser criteria, 23 on the original McDonald criteria, 19 on the 2005 revised McDonald criteria, 16 on the 2010 revised McDonald criteria, and 17 on the 2017 revised McDonald criteria.^25–29^ The inclusion criteria for the current analyses were that the patients had undergone [^11^C]PK11195 PET imaging with the ECAT High Resolution Research Tomograph (HRRT) scanner to detect brain glial cell activation, MRI to provide anatomical reference and to evaluate pathology related to MS, clinical assessment including EDSS score determination, and a blood sample obtained within 180 days of PET imaging. The exclusion criteria were intolerance to PET or MRI, pregnancy, other significant CNS pathology besides MS, and a relapse within 2 months prior to imaging. Healthy controls (HC) with no known neurological symptoms or diseases were selected to match the age and sex of the included patients. All participants gave their informed consent before participation.

Clinical assessment was conducted by an experienced clinician to evaluate the EDSS score using a standardized examination form (neurostatus.net). Multiple Sclerosis Severity Score was determined, body mass and height were measured, and body mass index (BMI) calculated as mass (kg)/height (m)^2^. The annualized relapse rate was calculated as the number of relapses/disease duration in years.

### Soluble marker assessments

Venous blood samples were collected, and serum was stored at −80°C within 2 h of sampling as previously reported.^30^ Concentrations of sGFAP and serum neurofilament light (sNfL) were measured in duplicates (mean coefficients of variation 6.9% and 4.7%, respectively) using the Neurology 2 plex B assay (Quanterix) and single molecule array (Simoa®) technology at the University Hospital and University of Basel, Basel, Switzerland.

### TSPO-positron emission tomography

PET scans of patients with MS and HCs were performed with a brain-dedicated ECAT HRRT scanner (CTI/Siemens, Knoxville, TN, USA) as previously described.^31^ The [^11^C]PK11195-radioligand was synthesized according to previously described methodology.^32^ A 60-min dynamic PET scan was started simultaneously with an intravenous injection of the [^11^C]PK11195 radioligand. The mean (SD) injected dose of [^11^C]PK11195 was 478 (43) MBq for patients with MS and 495 (15) MBq for HCs (*p* = 0.18), respectively. Prior to the ligand infusion, a 6-min transmission scan for attenuation correction was obtained using a 137Cs point source. A thermoplastic mask was used to minimize head movement during the scanning. Intake of benzodiazepines was prohibited since the previous evening before imaging.

PET images were reconstructed using 17 time frames as previously described.^31,33,34^ All images were corrected for decay, attenuation, scattering, random events, scanner dead time, and detector sensitivity.^34^ The reconstructed PET images were smoothed using a Gaussian 2.5 mm post-reconstruction filter.^33^ Possible displacements between frames were corrected using mutual information realignment with SPM8 software. Finally, PET images were coregistered to T1 MRI and resampled to match the MRI voxel size of 1 mm × 1 mm × 1 mm. Innate immune cell activation was evaluated as specific binding of [^11^C]PK11195 using the distribution volume ratio (DVR) in prespecified regions of interest (ROIs). For the estimation of the [^11^C]PK11195 DVR, the time-activity curve corresponding to a reference region devoid of specific TSPO-binding was acquired for each PET session using a supervised clustering algorithm with four predefined kinetic tissue classes using SuperPK software.^35,36^ The reference tissue input Logan method with a time interval from 20 to 60 min was applied to the regional time-activity curves using the supervised cluster algorithm gray matter (GM) reference input.^37^

For the individual lesion DVR analysis, the voxel-wise parametric binding potential (BP_ND_) maps were calculated as previously described.^31^ The resulting parametric maps were normalized to Montreal Neurological Institute space (MNI database) in SPM8 and the BP_ND_ images were transformed to DVR (DVR = BP_ND_ + 1). Active voxel threshold (DVR > 1.56) was defined as follows: First mean DVR + 1.96 × SD (95% confidence interval threshold) was calculated for each HC, and the average of these values was applied.^31^ Clusters below three connected active voxels were omitted to prevent the inclusion of random peak values. The T1 lesions were phenotyped as rim-active, overall-active, or inactive based on the proportion of active voxels in the lesion core versus the 2 mm rim as previously described.^31^ The number and volumes of rim-active and overall-active lesions were summed up and presented as the number and volume of TSPO-expressing lesions.

### Magnetic resonance imaging

MRI was performed with a 3T Ingenuity TF PET/MR System or a 3T Ingenia (Philips, Eindhoven, Netherlands) scanner (*n* = 45 and 35 people with MS; and 6 and 5 HCs, respectively) as previously described.^31,38^ The MRI protocol included T1-, T2-weighted, fluid-attenuated inversion recovery, and gradient echo (GRE, *n* = 73) sequences both to evaluate MS pathology and to acquire anatomic references for the PET image analysis. In addition, gadolinium-enhanced 3D-T1 was conducted for 56 people with MS. A manually edited semi-automated method was used to draw the combined T2 lesion ROI and combined T1 lesion ROI cores using Lesion Segmentation Tool (LST) in SPM8 (www.statistical-modelling.de/lst.html) and Carimas software (https://turkupetcentre.fi/carimas/) as previously described.^31^ The T1 lesion rim was obtained by dilating the combined T1 core mask by 2 mm and then removing the lesion core mask. The perilesional area was defined as the area 2–6 mm around the lesions. White matter (WM), NAWM, GM, and thalamus were segmented with the T1 lesion-filled T1 MRI (LST lesion-filling tool in SPM8) with the FreeSurfer 7.3.0 software (http://surfer.nmr.mgh.harvard.edu/) as previously described.^31^ T1 and T2 lesion loads in cm^3^ were calculated from the lesion ROI masks. The volumes of whole brain, NAWM, cortical GM, and thalamus were calculated from the FreeSurfer masks.

Paramagnetic rim lesions (PRL) were identified with Quantitative Susceptibility Mapping (QSM). QSM was acquired using a 3D flow-compensated GRE sequence with 60 slices, 2-mm thickness, acquisition matrix 240 mm × 187 mm (Ingenia) or 240 mm × 183 mm (Ingenuity), FOV = 240 mm × 184.8 mm (Ingenia) or 240 mm × 184 mm (Ingenuity), reconstructed voxels of 0.6 mm × 0.6 mm × 2 mm, FA = 15, TE/TR = 5.7/55 ms and AT = 3:49 (Ingenia) or 5:10 (Ingenuity) mm:ss. The QSM images were reconstructed using the Morphology Enabled Dipole Inversion (MEDI) toolbox with automatic uniform CSF zero reference from the multi-echo gradient echo data.^39^ To be identified as a PRL, a lesion had to have a hyperintense bright rim relative to the lesion core, as determined by two experienced raters.^40^ Both complete and partial (>50% of lesion circumference) hyperintense rims were considered as paramagnetic rims. In case of disagreement, a third reviewer was consulted to reach a final consensus. Finally, the QSM masks were registered to the T1 space and matched with the T1 lesions.

### Statistical analyses

Differences between RRMS, progressive MS, MS, and HC groups were tested with one-way ANOVA, Tukey-Kramer HSD, Chi-square, pooled *t*-test, Wilcoxon Signed Rank Test, or Fisher’s exact test, when applicable. For nonparametric multiple comparisons, Steel-Dwass’ test was conducted. Associations between variables in the MS group were first examined with Spearman’s rank correlation (ρ), Pearson correlation coefficient (*r*), or Point-Biserial correlation (*r*_pb_), when applicable. The relevant associations were examined further with standard least squares regression models. Age and disease-modifying treatment (DMT) status were included as explanatory variables in all the models. DMT was included as a dichotomic variable (yes/no) defined as any DMT for MS during initial data collection or within the previous 2 months. All the models were also separately tested with additional variables, BMI and sex, but neither of these variables was significant in any of the models, and they did not improve the coefficient of determination (*R*^2^) or alter the interpretation of the results. Therefore, they were not included in the final models. In addition, the models were tested with disease type (RRMS/progressive MS) as an explanatory variable, but it was significant only in the models with the brain volume, or cortical GM volume, and age as explanatory variables. Possible multicollinearity was carefully assessed by the variance inflation factor (VIF) and preliminary correlation analyses.

Logarithmic (log10) or square root (Sqrt) transformations were performed when necessary to achieve normal distribution of the residuals. The normal distributions of the residuals were examined visually and by the Shapiro-Wilk Test for Normality. Missing data was handled by pairwise deletion. If not otherwise stated, data are expressed as mean (SD). Nonlesional ROI volumes are presented as parenchymal fractions (PF). The level of statistical significance was set at 5%. In the correlation analyses, the level of statistical significance was set at 1% in order to correct for multiple comparisons. The correlation analyses were carried out with IBM SPSS Statistics 29.0.2.0 (IBM Corp., Armonk, NY, USA). All the other analyses were carried out with JMP Pro 17.0.0 for Windows (SAS Institute Inc., Cary, NC, USA).

## Results

### Characteristics of the study cohorts

The study cohort consisted of 80 people with MS and 11 HCs (Table 1). The ethnicity of the HCs and 79 MS patients was white European, one patient had North African origin. The mean (SD) age of the participants was 45.5 (7.6) years. The age difference between all MS patients (45.9 (7.6) years) and the HC group (42.2 (6.5) years) was not significant (*p* = 0.13). However, the people with progressive MS (51.9 (9.2) years) were older than HCs and people with RRMS (44.7 (6.7) years; Table 1). Out of the included people with MS, 66 had RRMS, 11 had secondary progressive MS, and 3 had primary progressive MS according to the 2017 revised McDonald criteria.^29^ Twenty-eight of the 80 people with MS had no DMT at the time or within 2 months prior to data collection. Nineteen participants were treated with teriflunomide, 8 with fingolimod, 5 with dimethyl fumarate, 5 with glatiramer acetate, 5 with interferon beta-1a, 1 with peginterferon beta-1a, 6 with rituximab, 2 with natalizumab, and 1 with cladribine. Twenty-eight participants had DMT modifications during the last 12 months; 11 had treatment cessation on average 3.1 (2.7) months prior to data collection, and 17 participants had a new DMT initiated on average 5.3 (3.7) months before data collection. The median (Q1, Q3, min–max) duration between blood sampling and PET imaging was 7 (0, 35, 0–154) days.

**Table 1.** Characteristics of the study cohort.

Variable	HC	MS	RRMS	PMS	HC vs MS *p*	HC vs RRMS *p*	HC vs PMS *p*	RRMS vs PMS *p*
*n*	11	80	66	14				
Women, *n* (%)	8 (72.7)	55 (68.8)	47 (71.2)	8 (57.1)	1.0	0.92	0.42	0.31
Age, years	42.2 (6.5)	45.9 (7.6)	44.7 (6.7)	51.9 (9.2)	0.13	0.54	0.0029	0.0022
BMI, kg/m^2^ ^a^	24.9 (23.0, 26.8)^b^	26.8 (22.7, 30.9)^b^	26.8 (22.9, 31.4)	26.8 (21.4, 30.8)	0.23	ns	ns	ns
Disease duration, years^c^	na	13.6 (8.4)	12.4 (7.9)	19.1 (8.9)	na	na	na	0.0063
DMT, *n* (%)	na	52 (65)	46 (69.7)	6 (42.9)	na	na	na	0.070
EDSS^b^	na	2.9 (1.6)	2.4 (1.0)	5.3 (1.4)	na	na	na	<0.0001
MSSS^d^	na	3.3 (1.6, 5.0)	2.6 (1.4, 4.6)	4.6 (4.2, 7.7)	na	na	na	0.0007
ARR^a,e^	na	0.3 (0.2, 0.5)	0.3 (0.2, 0.6)	0.3 (0.1, 0.3)	na	na	na	0.24
Time from last relapse, years^b,e^	na	3.5 (1.1, 8.6)	2.6 (0.9, 7.7)	7.9 (3.3, 13.4)	na	na	na	0.028
Gd+, *n* (%)^f^	na	6 (11)	5 (11)	1 (9)	na	na	na	1.0
sNfL, pg/ml^a^	8.2 (4.4, 9.3)	9.4 (7.1, 15.0)	8.7 (6.9, 13.9)	14.2 (9.2, 22.1)	0.023	0.13	0.0027	0.036
sGFAP, pg/ml^a^	59.0 (41.1, 86.7)	91.6 (65.2, 121.1)	77.1 (64.2, 116.4)	122.0 (90.7, 164.8)	0.0083	0.062	0.0002	0.0056
Whole brain volume, PF%^d^	86.7 (85.0, 88.7)^b^	83.4 (80.4, 89.5)^b^	84.4 (81.7, 86.9)	79.5 (76.1, 83.7)	0.011	0.11	0.0012	0.017
NAWM volume, PF%^d^	35.8 (33.9, 36.5)^b^	33.6 (31.1, 35.1)^b^	33.8 (32.3, 35.7)	29.9 (27.7, 31.4)	0.015	0.17	<0.0001	<0.0001
Cortical GM volume, PF%^g^	33.7 (32.5, 34.5)	31.8 (30.4, 33.0)	31.9 (31.1, 33.1)	29.6 (27.6, 31.3)	0.017	0.017	0.0095	0.013
Thalamus volume, PF%^g^	1.13 (1.09, 1.15)^b^	1.02 (0.92, 1.09)^b^	1.04 (0.97, 1.09)	0.80 (0.79, 1.00)	0.0004	0.0044	0.0003	0.0003

The data are presented as mean (SD) unless otherwise stated. The differences between the three groups, that is, RRMS, PMS, and HC, were tested with one-way ANOVA and Tukey-Kramer HSD, Steel-Dwass’ test, or Chi-square approximation, when applicable. The differences between the two groups, that is, MS versus HC and RRMS versus PMS, when the analysis was not applicable to healthy participants, were tested with a pooled *t*-test, Wilcoxon Signed Rank Test, or Fisher’s exact test, when applicable.

a Presented as median (Q1, Q3) and analysis conducted with log10-transformed estimates.

b Comparison between two groups conducted with Wilcoxon Signed Rank Test.

c Disease duration from first symptoms to data collection.

d Presented as median (Q1, Q3) and analysis conducted with square root-transformed estimates.

e Data missing from three participants with primary progressive MS.

f Data available from 46 participants with RRMS and 11 participants with PMS, respectively.

g Comparison between three groups conducted with Steel-Dwass’ test.

ARR, annualized relapse rate; BMI, body mass index; DMT, disease-modifying treatment; EDSS, Expanded Disability Status Scale; Gd+, positive finding in gadolinium-enhanced magnetic resonance imaging; GM, gray matter; HC, healthy control; MS, multiple sclerosis; MSSS, MS Severity Score; na, not applicable; NAWM, normal-appearing white matter; ns, ANOVA non-significant; PF, parenchymal fraction; PMS, progressive multiple sclerosis; RRMS, relapsing-remitting multiple sclerosis; sGFAP, serum glial fibrillary acid protein; sNfL, serum neurofilament light.

**Figure 1.**
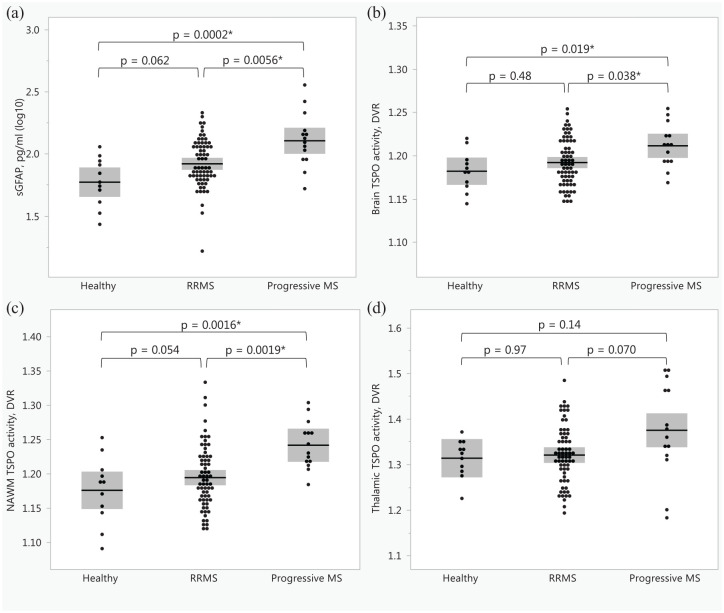
The differences between healthy participants and people with relapsing-remitting multiple sclerosis and progressive multiple sclerosis in baseline serum glial fibrillary acid protein (a) and in TSPO-PET measured inflammation in whole brain (b), normal-appearing white matter (c) and thalamus (d). The differences in panels (a), (b), and (c) were tested with one-way ANOVA and Tukey-Kramer HSD. The differences in panel (d) were tested with Steel-Dwass’ test. *Indicates a statistically significant difference; the shaded area represents standard deviation. DVR, distribution volume ratio; MS, multiple sclerosis; NAWM, normal-appearing white matter; PET, positron emission tomography; RRMS, relapsing-remitting multiple sclerosis; sGFAP, serum glial fibrillary acid protein; TSPO, 18kDa translocator protein.

People with progressive MS had higher sGFAP (122.0 (90.7, 164.8) pg/ml) compared to HCs (59.0 (41.1, 86.7) pg/ml) and people with RRMS (77.1 (64.2, 116.4) pg/ml; Table 1, Figure 1). People with progressive MS also had higher whole brain and NAWM DVR (1.21 (0.03), 1.24 (0.04), respectively) compared to HCs (1.18 (0.02), 1.18, (0.05), respectively) and people with RRMS (1.19 (0.03), 1.19 (0.05), respectively; Figure 1). People with progressive MS had higher numbers and volumes of MS lesions compared to RRMS (Table 2). Only the number and volume of TSPO-inactive lesions did not differ between MS groups (Table 2).

**Table 2.** Differences in MS lesion characteristics between people with relapsing-remitting MS and progressive MS.

V﻿ariable	MS	RRMS	PMS	RRMS vs PMS *p*
*n*	80	66	14	
T1 lesion load, cm^3 a^	2.8 (1.4, 8.1)	2.1 (1.2, 4.7)	15.8 (10.4, 30.8)	<0.0001
T2 lesion load, cm^3 b^	5.6 (2.8, 16.3)	3.9 (2.7, 9.5)	23.7 (17.6, 37.7)	<0.0001
Number of T1 WM lesions^b^	15 (8, 24)	12 (6, 21)	30 (20, 44)	0.0002
Number of TSPO-expressing lesions^b^	8 (3, 16)	7 (2, 13)	23 (14, 34)	<0.0001
Number of TSPO-inactive lesions^c^	6 (3, 8)	6 (3, 8)	7 (5, 9)	0.11
Volume of TSPO-expressing lesions, cm^3 b^	1.3 (0.4, 6.5)	1.1 (0.3, 2.8)	14.6 (6.5, 26.9)	<0.0001
Volume of TSPO-inactive lesions, cm^3 c^	0.5 (0.2, 0.8)	0.5 (0.2, 0.8)	0.5 (0.4, 0.9)	0.18
Number of PRLs^b,d^	0 (0, 2)	0 (0, 1)	2 (1, 5)	0.0012
Volume of lesions with PRLs, cm^3 b,d^	0 (0, 1.1)	0 (0, 0.6)	12.2 (2.1, 23.3)	<0.0001

The data are presented as median (Q1, Q3). Unless otherwise stated, the differences between groups were tested with pooled *t*-tests, when applicable.

a Analysis conducted with log10-transformed estimates.

b Analysis conducted with Wilcoxon Signed Rank Test.

c Analysis conducted with square root-transformed estimates.

d Data available from 62 participants with RRMS and 11 participants with progressive MS, respectively.

MS, multiple sclerosis; PMS, progressive MS; PRL, paramagnetic rim lesion; RRMS, relapsing-remitting MS; TSPO, 18 kDa translocator protein; WM, white matter.

### Associations between sGFAP and clinical parameters

Among people with MS, sGFAP (log10) correlated positively with age at data collection (*r* = 0.30, *p* = 0.0075), but not with BMI (log10; *r* = −0.18, *p* = 0.10). There was no statistically significant difference in sGFAP (log10) between men (median 91.1 pg/ml) and women (median 92.1 pg/ml, *p* = 0.41, Supplemental Material—Additional Table 1) or between treated (median 77.7 pg/ml) and untreated patients (median 108.2 pg/ml, *p* = 0.057). sGFAP did not correlate with EDSS (ρ = 0.16, *p* = 0.15), disease duration (ρ = 0.06, *p* = 0.61), or the time from last relapse (ρ = 0.18, *p* = 0.12), nor sGFAP (log10) with the ARR (log10; *r* = −0.02, *p* = 0.86). However, sGFAP (log10) correlated positively with sNfL (log10; *r* = 0.60, *p* < 0.0001). The differences in soluble biomarkers and DVRs between men and women are presented in Supplemental Material—Additional Table 1.

### Correlations between sGFAP and MRI and PET outcomes

**Figure 2.**
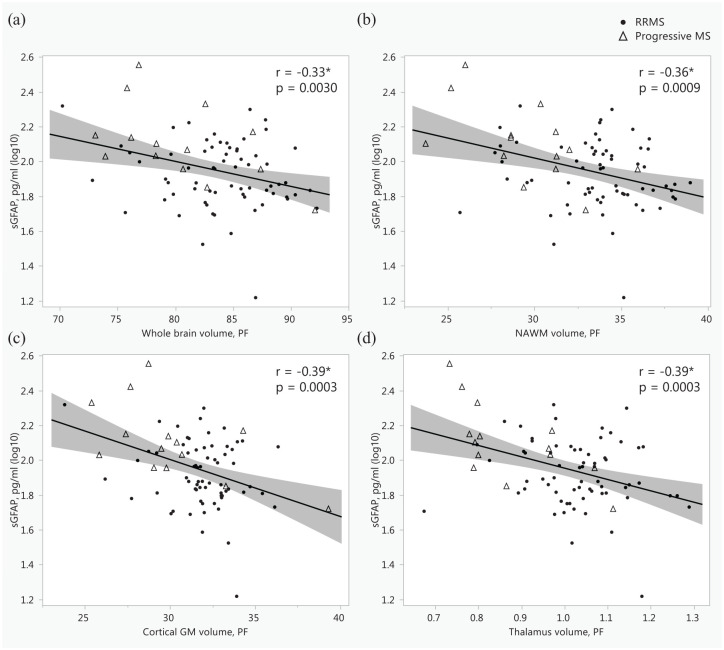
Associations between serum glial fibrillary acid protein and the volumes of whole brain (a), normal-appearing white matter (b), cortical gray matter (c), and thalamus (d), presented as scatterplots with Pearson correlation coefficients. *Indicates a statistically significant association; the shaded area represents the 95% confidence interval. GM, gray matter; MS, multiple sclerosis; NAWM, normal-appearing white matter; PF, parenchymal fraction, %; RRMS, relapsing-remitting MS; sGFAP, serum glial fibrillary acid protein.

**Figure 3.**
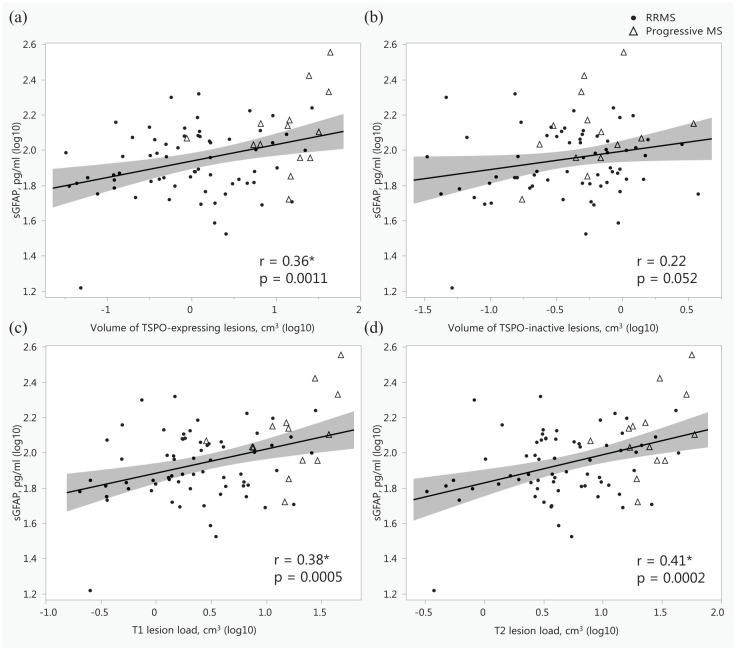
Associations between serum glial fibrillary acid protein and the volume of TSPO-expressing white matter lesions (a), the volume of TSPO-inactive white matter lesions (b), T1 lesion load (c), and T2 lesion load (d), presented as scatterplots with Pearson correlation coefficients. *Indicates a statistically significant association; the shaded area represents the 95% confidence interval. MS, multiple sclerosis; RRMS, relapsing-remitting MS; sGFAP, serum glial fibrillary acid protein; TSPO, 18 kDa translocator protein.

Among people with MS, higher sGFAP correlated with lower whole brain, NAWM, cortical GM, and thalamus volumes (Figure 2). In addition, higher sGFAP correlated with increased T1 and T2 lesion loads (Figure 3). However, the number of PRLs or the volume of lesions with PRLs was not associated with sGFAP (ρ = 0.10, 0.17; *p* = 0.40, 0.16, respectively). MRI data with contrast agent gadolinium were available from 45 patients with RRMS and 11 patients with progressive MS, respectively. The presence of gadolinium-enhancing lesions was associated with higher sNfL (log10; *r*_pb_ = 0.38, *p* = 0.0040), but not with sGFAP (log10; *r*_pb_ = 0.13, *p* = 0.32). Similarly, the volume of gadolinium-enhancing lesions was associated with higher sNfL (ρ = 0.34, *p* = 0.010), but not with sGFAP (ρ = 0.12, *p* = 0.38). Therefore, the participants with gadolinium-positive lesions were not excluded from these analyses.

**Figure 4.**
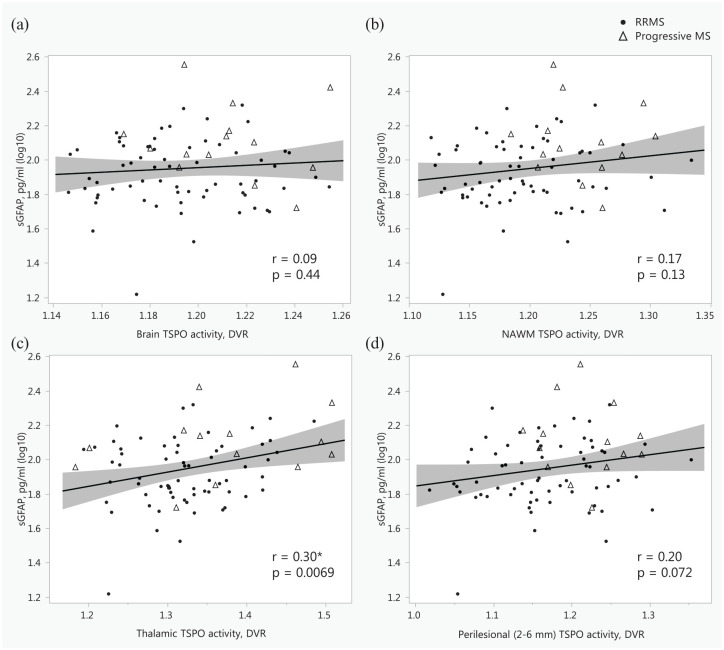
Associations between serum glial fibrillary acid protein and TSPO-PET measured innate immune cell activation in whole brain (a), normal-appearing white matter (b), thalamus (c), and perilesional area 2–6 mm around lesions (d), presented as scatterplots with Pearson correlation coefficients. *Indicates a statistically significant association; the shaded area represents the 95% confidence interval. DVR, distribution volume ratio; MS, multiple sclerosis; NAWM, normal-appearing white matter; PET, positron emission tomography; RRMS, relapsing-remitting MS; sGFAP, serum glial fibrillary acid protein; TSPO, 18kDa translocator protein.

Higher sGFAP correlated with increased thalamus DVR and percentage of TSPO-active voxels in thalamus (*r* = 0.33, *p* = 0.0023), but not with TSPO-binding in the NAWM (Figure 4). There were no correlations between sGFAP and distinctive cortical regions or other investigated subcortical regions (Supplemental Material—Additional Table 2). Additionally, higher sGFAP correlated with increased volume of TSPO-expressing lesions (Figure 3).

Correlations were additionally examined separately in RRMS and progressive MS patients. Generally, no diverging associations or data interpretations were found. However, among patients with progressive MS (*n* = 14), BMI correlated inversely with sGFAP (log10; *r* = −0.60, *p* = 0.022). The correlation coefficients in the RRMS and progressive MS groups regarding the variables presented in Figures 2–4 are presented in Supplemental Material—Additional Table 3.

### Associations between sGFAP and imaging variables assessed with regression modeling

Standard least squares regression models were created to further evaluate the associations between sGFAP and imaging variables of interest. All the models were adjusted with possible confounding factors, age and DMT, which together explained 13% of the variation in sGFAP (Supplemental Material—Additional Table 4). The results of the models with lesion-related parameters predicting sGFAP, adjusted with age and DMT, are presented in Table 3. The models including the volume of TSPO-expressing lesions or T1 or T2 lesion loads as explanatory variables, were the best predictors of sGFAP, explaining 27% of the variation in sGFAP (Table 3).

**Table 3.** The results from standard least squares regression models predicting serum glial fibrillary acid protein (log10) with lesion characteristics as explanatory variables.

Model variables	β	VIF	DF	*F* ratio	*p*	*R* ^2^
Volume of TSPO-expressing lesions, cm^3^	0.39	1.04	1	15.2	0.0002	0.27
Age at sampling, years	0.21	1.04	1	4.4	0.040	
DMT [0]	0.18	1.00	1	3.5	0.065	
Volume of TSPO-inactive lesions, cm^3^	0.06	1.04	1	0.3	0.61	0.13
Age at sampling, years	0.28	1.01	1	6.8	0.011	
DMT [0]	0.19	1.03	1	3.0	0.088	
T1 lesion load, cm^3^	0.39	1.05	1	15.0	0.0002	0.27
Age at sampling, years	0.21	1.05	1	4.2	0.044	
DMT [0]	0.18	1.01	1	3.4	0.070	
T2 lesion load, cm^3^	0.38	1.05	1	14.6	0.0003	0.27
Age at sampling, years	0.21	1.05	1	4.2	0.045	
DMT [0]	0.18	1.00	1	3.5	0.065	
Volume of lesions with PRLs, cm^3^ (sqrt)^a,b^	0.38	1.00	1	13.0	0.0006	0.24
Age at sampling, years	0.26	1.00	1	6.2	0.015	
DMT [0]	0.17	1.00	1	2.8	0.10	

Age and the presence of disease-modifying treatment were included as explanatory variables in all the models.

a Data available from 62 participants with RRMS and 11 participants with progressive MS, respectively.

b The model had one extreme outlier in the residuals. A sensitivity analysis was conducted by excluding this outlier; the model reached *R*^2^ 0.21 and the interpretation of the results was not affected.

DF, degrees of freedom; DMT [0], no disease-modifying treatment; No., number; PRL, paramagnetic rim lesion; TSPO, 18 kDa translocator protein; VIF, variance inflation factor.

The models with TSPO-PET and MRI volumetric parameters predicting sGFAP, adjusted with age and DMT, are presented in Table 4. The percentage of TSPO-active voxels in the thalamus, together with age and DMT status, explained 24% of the variation in sGFAP (Table 4). Thalamus volume, together with age and DMT status, similarly explained 24% of the variation in sGFAP (Table 4). However, age lost its significance in the models with brain volumetric variables as explanatory variables, most probably due to multicollinearity.

**Table 4.** The results from standard least squares regression models predicting serum glial fibrillary acid protein (log10) with TSPO activity or brain volumetric parameters as explanatory variables.

Model variables	β	VIF	DF	*F* ratio	*p*	*R* ^2^
Thalamic TSPO activity, DVR	0.33	1.03	1	10.3	0.0019	0.23
Age at sampling, years	0.27	1.01	1	7.1	0.009	
DMT [0]	0.25	1.03	1	5.9	0.018	
% of TSPO-active voxels in thalamus	0.35	1.06	1	11.6	0.0011	0.24
Age at sampling, years	0.23	1.03	1	5.1	0.026	
DMT [0]	0.26	1.04	1	6.7	0.012	
Brain TSPO activity, DVR	0.06	1.25	1	0.2	0.62	0.13
Age at sampling, years	0.26	1.16	1	5.3	0.024	
DMT [0]	0.21	1.11	1	3.6	0.062	
NAWM TSPO activity, DVR	0.14	1.17	1	1.5	0.23	0.14
Age at sampling, years	0.24	1.13	1	4.5	0.037	
DMT [0]	0.23	1.06	1	4.3	0.042	
Perilesional TSPO activity, DVR	0.17	1.06	1	2.3	0.13	0.15
Age at sampling, years	0.25	1.06	1	5.2	0.026	
DMT [0]	0.21	1.01	1	4.0	0.048	
Thalamus volume, PF	−0.36	1.15	1	11.0	0.0014	0.24
Age at sampling, years	0.16	1.14	1	2.2	0.14	
DMT [0]	0.23	1.01	1	5.2	0.025	
Whole brain volume, PF	−0.26	1.15	1	5.3	0.024	0.18
Age at sampling, years	0.19	1.16	1	2.9	0.091	
DMT [0]	0.20	1.00	1	3.7	0.057	
NAWM volume, PF	−0.31	1.07	1	8.5	0.0046	0.21
Age at sampling, years	0.21	1.07	1	4.0	0.050	
DMT [0]	0.19	1.00	1	3.6	0.062	
Cortical GM volume, PF	−0.34	1.37	1	8.1	0.0058	0.21
Age at sampling, years	0.11	1.37	1	0.8	0.37	
DMT [0]	0.22	1.01	1	4.4	0.039	

Age and the presence of disease-modifying treatment were included as explanatory variables in all the models.

DF, degrees of freedom; DMT [0], no disease-modifying treatment; DVR, distribution volume ratio; GM, gray matter; NAWM, normal-appearing white matter; PF, parenchymal fraction; TSPO, 18 kDa translocator protein; VIF, variance inflation factor.

In addition, we wanted to test whether the *R*^2^ could be improved with the addition of a lesion-related parameter and a parameter describing thalamic TSPO-binding in the same model. The best predictors (with highest *R*^2^ and *F*-ratios) from the models with three explanatory variables, that is, volume of TSPO-expressing lesions/T1/T2 lesion loads on the one hand, and on the other hand thalamus DVR/percentage of TSPO-active voxels in thalamus, were chosen to a regression model with four explanatory variables. The highest *R*^2^ and smallest VIFs were reached with the model including the volume of TSPO-expressing lesions, thalamus DVR, age at data collection, and DMT as explanatory variables; together explaining 29% of the variation in sGFAP (Table 5). Thalamus DVR was no longer significant in the model, but it improved the *R*^2^ and thus was kept in the final model. However, all the tested models, which fulfilled the normal distribution assumption of the residuals (i.e., models including T1 or T2 lesion load, thalamic TSPO activity, age, and DMT), reached a similar conclusion (Supplemental Material—Additional Table 5).

**Table 5.** The results from a standard least squares regression model predicting serum glial fibrillary acid protein (log10) with four explanatory variables.

Model variables	β	VIF	DF	*F* ratio	*p*	*R* ^2^
Volume of TSPO-expressing lesions, cm^3^	0.30	1.40	1	6.8	0.011	0.29
Thalamic TSPO activity, DVR	0.18	1.38	1	2.4	0.13	
Age at sampling, years	0.22	1.05	1	4.8	0.031	
DMT [0]	0.21	1.05	1	4.7	0.034	

DF, degrees of freedom; DMT [0], no disease-modifying treatment; DVR, distribution volume ratio; TSPO, 18 kDa translocator protein; VIF, variance inflation factor.

Similarly, adjusting the association between sGFAP and thalamus volume with T2 lesion load or the volume of TSPO-expressing lesions, thalamus volume was no longer significant in the model (Supplemental Material—Additional Table 6). In addition, thalamus volume correlated strongly with the volume of TSPO-expressing lesions (Supplemental Material—Additional Figure 1). Moreover, NAWM and thalamus volumes and TSPO activity correlated inversely (Supplemental Material—Additional Figure 2). In a regression model with thalamus volume (PF) as the dependent variable, NAWM and thalamus TSPO activity, together with sGFAP and age, explained 54% of the variation in thalamus PF (Supplemental Material—Additional Table 7).

When the regression models were tested with disease type (RRMS/progressive MS) as an explanatory variable, it was significant only in the models with the brain volume or cortical GM volume as explanatory variables (Supplemental Material—Additional Table 8). However, it did not significantly strengthen the associations between brain volumetrics and sGFAP.

## Discussion

In the current study, we show that among patients with MS, sGFAP associates with the volume of WM lesions, evaluated with either T1- or T2-weighted MRI. In addition, sGFAP associates with chronic TSPO-expressing MS lesions. Blood-soluble GFAP is derived from a multitude of sources, and our work demonstrates that in the currently studied cohort, 27% of the variation in sGFAP was explained by the volume of TSPO-expressing lesions, age, and DMT. While PET imaging of MS patients is not feasible in routine clinical practice for assessment of glial activation, our results suggest that sGFAP could serve as an indicator of ongoing glial activation, particularly within chronic WM lesions.

### Associations of sGFAP with lesion characteristics

In the current study, sGFAP was significantly associated with the T1 and T2 lesion loads (Figure 3), a finding in accordance with previous work.^9,41^ In addition, sGFAP is associated with the volume of TSPO-expressing lesions. TSPO-radioligands bind to both microglia and astrocytes in the MS brain, so when performing imaging only using a TSPO-targeting ligand, it is impossible to define the exact cellular source responsible for increased radioligand binding. Neuropathological work has, however, demonstrated a dense astrocytic scar in the core of chronic MS lesions,^42,43^ and it is possible that TSPO expression by astrocytes is partly responsible for the enhanced TSPO signal in the chronic T1 (TSPO-expressing) lesions. Therefore, given the association between sGFAP and chronic TSPO-expressing lesions, it is plausible to hypothesize that astrocytes in the chronic lesions are a potential partial source of the elevated sGFAP. We are currently performing detailed histological studies on human postmortem MS brain to address this question further.

Interestingly, in marmoset experimental autoimmune encephalomyelitis (EAE), a preclinical primate model of MS, TSPO expression was present in GFAP-positive astrocytes in lesions with astrogliosis, but not in lesions without astrogliosis.^44^ In human MS brain tissue, hypertrophied astrocytes are present in several MS lesion types, including histologically mixed active/inactive lesions, and an astrocytic scar is generally found in inactive lesions.^45^ Moreover, upregulation of both GFAP and TSPO expression has been observed in inflamed astrocytes as well as in activated microglia at the edge of chronic active lesions.^46,47^ In the marmoset EAE, TSPO expression was not present in astrocytes in the NAWM,^44^ and in the present study, NAWM TSPO activity did not associate with sGFAP.

In accordance with a previous study, we did not observe a correlation between sGFAP and the volume of lesions with PRLs.^48^ However, assessed with a regression analysis, the volume of lesions with PRLs, together with age and DMT, explained 24% of the variation in sGFAP. In addition, in the current study, the volume of gadolinium-enhancing lesions was associated only with sNfL but not with sGFAP. This is in line with previous studies^49,50^ and supports the conception that sNfL elevation and sGFAP elevation are driven by different pathological mechanisms, with sNfL at least partly reflecting acute focal inflammatory activity with increased blood-brain-barrier permeability and focal lesion-associated axonal damage, while sGFAP may reflect reactive astrogliosis associated with PIRA.

### Associations with thalamic TSPO activity and thalamic volume

Thalamic atrophy occurs early in MS and the rate of atrophy remains consistent throughout the disease course.^51^ However, thalamic pathology in MS is not limited to atrophy, but extends to heterogeneous microstructural alterations that relate to cognitive disability progression.^52,53^ Moreover, thalamic TSPO activity predicts disability progression in MS.^19^ In the current study, sGFAP was associated with both thalamic TSPO activity and volume.

WM lesions are considered to contribute to thalamic degeneration.^53,54^ Therefore, the association between sGFAP and thalamic pathology likely closely relates to the magnitude of the WM lesion burden. This viewpoint is supported by the finding that in regression analyses, thalamic TSPO activity was no longer significant when adjusted for the volume of TSPO-expressing lesions or T1 or T2 lesion loads. However, particularly early thalamic atrophy (even in the absence of WM lesions) can be triggered by silent microstructural thalamic alterations and degeneration of thalamic tracts measured by DTI.^55,56^ Interestingly, increased NAWM TSPO activity correlated strongly with decreased thalamus volume in the current study (Supplemental Figure 2). In a regression model with thalamus volume (PF) as the dependent variable, NAWM and thalamus TSPO activity, together with sGFAP and age, explained 54% of the variation in thalamus PF, with a statistically significant contribution from all variables, excluding age (Supplemental Material—Additional Table 4). Previously, associations between increased NAWM TSPO activity and brain atrophy have been reported.^20,57–59^ Therefore, it can be hypothesized that diffuse smoldering inflammation in the NAWM could cause thalamic atrophy by deranging the function of thalamocortical tracts, but the current cross-sectional association determination cannot confirm a causal relation. Along this cascade of events, the smoldering inflammation in the NAWM and thalamus could gradually lead to thalamic atrophy, and over time, the sustained high amount of reactive astrocytes in and around the active lesions, as well as in the thalamus, could be reflected as elevated sGFAP.

### Strengths and limitations

The simultaneous application of state-of-the-art analysis methods addressing CNS pathology in MS, that is, *in vivo* measurement of glial activity using PET imaging and the application of ultra-sensitive biomarker detection to analyze blood sGFAP, can be considered strengths of the current study.

On the other hand, the lack of a positive control group with another neurological disease can be considered a limitation. Additionally, the time interval between PET imaging and blood sampling could have been shorter in some cases. However, 50% of the patients had the blood sample obtained within a week of imaging, and 75% of patients had the blood sample and PET imaging obtained within 35 days. Furthermore, multicollinearity is an important issue that needs to be considered when interpreting regression results. Although the VIFs remained below 2 in the final reported regression models, the finding that age lost its significance when brain volumetric outcomes were added to the same model indicates that the included variables partly measure the same features. Therefore, the *R*^2^ in these models may be inaccurate.

## Conclusion

Blood-soluble GFAP is derived from a multitude of sources, and T2 lesion burden is a significant known contributor to higher sGFAP among people with MS. In the current study, we explored the association between sGFAP and glial activity. Particularly, the volume of TSPO-expressing lesions correlated with the blood sGFAP concentration, which suggests that the elevated sGFAP may partly reflect astrogliosis in the chronic WM lesions. As reported earlier, sGFAP associates with MS disease progression and adverse imaging outcomes. The finding of the association between a high prevalence of TSPO-expressing WM lesions and high sGFAP suggests that lesion-associated glial activity may promote MS progression partially via astrocyte-driven mechanisms. A combination of various soluble biomarkers and PET ligands for specific cell types may add to the understanding of progression-promoting cellular mechanisms in the brain.

## Supplemental Material

sj-docx-1-tan-10.1177_17562864251352998 – Supplemental material for Serum glial fibrillary acid protein associates with TSPO-expressing lesions in multiple sclerosis brain Supplemental material, sj-docx-1-tan-10.1177_17562864251352998 for Serum glial fibrillary acid protein associates with TSPO-expressing lesions in multiple sclerosis brain by Tanja Sjöros, Maija Saraste, Markus Matilainen, Marjo Nylund, Mikko Koivumäki, Jens Kuhle, David Leppert and Laura Airas in Therapeutic Advances in Neurological Disorders

sj-pdf-2-tan-10.1177_17562864251352998 – Supplemental material for Serum glial fibrillary acid protein associates with TSPO-expressing lesions in multiple sclerosis brain Supplemental material, sj-pdf-2-tan-10.1177_17562864251352998 for Serum glial fibrillary acid protein associates with TSPO-expressing lesions in multiple sclerosis brain by Tanja Sjöros, Maija Saraste, Markus Matilainen, Marjo Nylund, Mikko Koivumäki, Jens Kuhle, David Leppert and Laura Airas in Therapeutic Advances in Neurological Disorders

## Declarations

**Ethics approval and consent to participate:** The study was conducted according to the Declaration of Helsinki and approved by the Ethics Committee of the Hospital District of Southwest Finland (decision numbers 19/1801/2016, 86/1800/2017, 67/1801/2018, 89/1800/2019). All participants gave their written informed consent before entering the study.

**Consent for publication:** Not applicable.

**Availability of data and materials:** The datasets used and/or analyzed during the current study are available from the corresponding author on reasonable request.
