# Supplementary material for: Serum glial fibrillary acid protein associates with TSPO-expressing lesions in multiple sclerosis brain
Source: Ther Adv Neurol Disord. 2025 Jul 28;18:17562864251352998. doi: 10.1177/17562864251352998 (PMC12314351; doi:10.1177/17562864251352998)
Supplement: sj-pdf-2-tan-10.1177_17562864251352998 – Supplemental material for Serum glial fibrillary acid protein associates with TSPO-expressing lesions in multiple sclerosis brain [file sj-pdf-2-tan-10.1177_17562864251352998.pdf]

Serum Glial Fibrillary Acid Protein Associates with TSPO-expressing Lesions in Multiple Sclerosis Brain

Tanja Sjöros[1,2,3], Maija Saraste[1,2,3,4], Markus Matilainen[1,2,3], Marjo Nylund[1,2,3,4], Mikko Koivumäki[1], Jens Kuhle[5,6], David Leppert[5,6], and Laura Airas[1,2,3,4]

1 Turku PET Centre, University of Turku, Åbo Akademi University, and Turku University Hospital, Turku, Finland

2 Clinical Neurosciences, University of Turku, Turku, Finland

3 InFLAMES Research Flagship, University of Turku, Turku, Finland

4 Neurocenter, Turku University Hospital, Turku, Finland

5 Department of Neurology, University Hospital and University of Basel, Basel, Switzerland

6 Departments of Biomedicine and Clinical Research, Multiple Sclerosis Centre and Research Center for Clinical Neuroimmunology and Neuroscience (RC2NB), University Hospital and University of Basel, Basel, Switzerland

Additional files:

Additional Figures 1–2.

Additional Tables 1–8.

Additional Figures

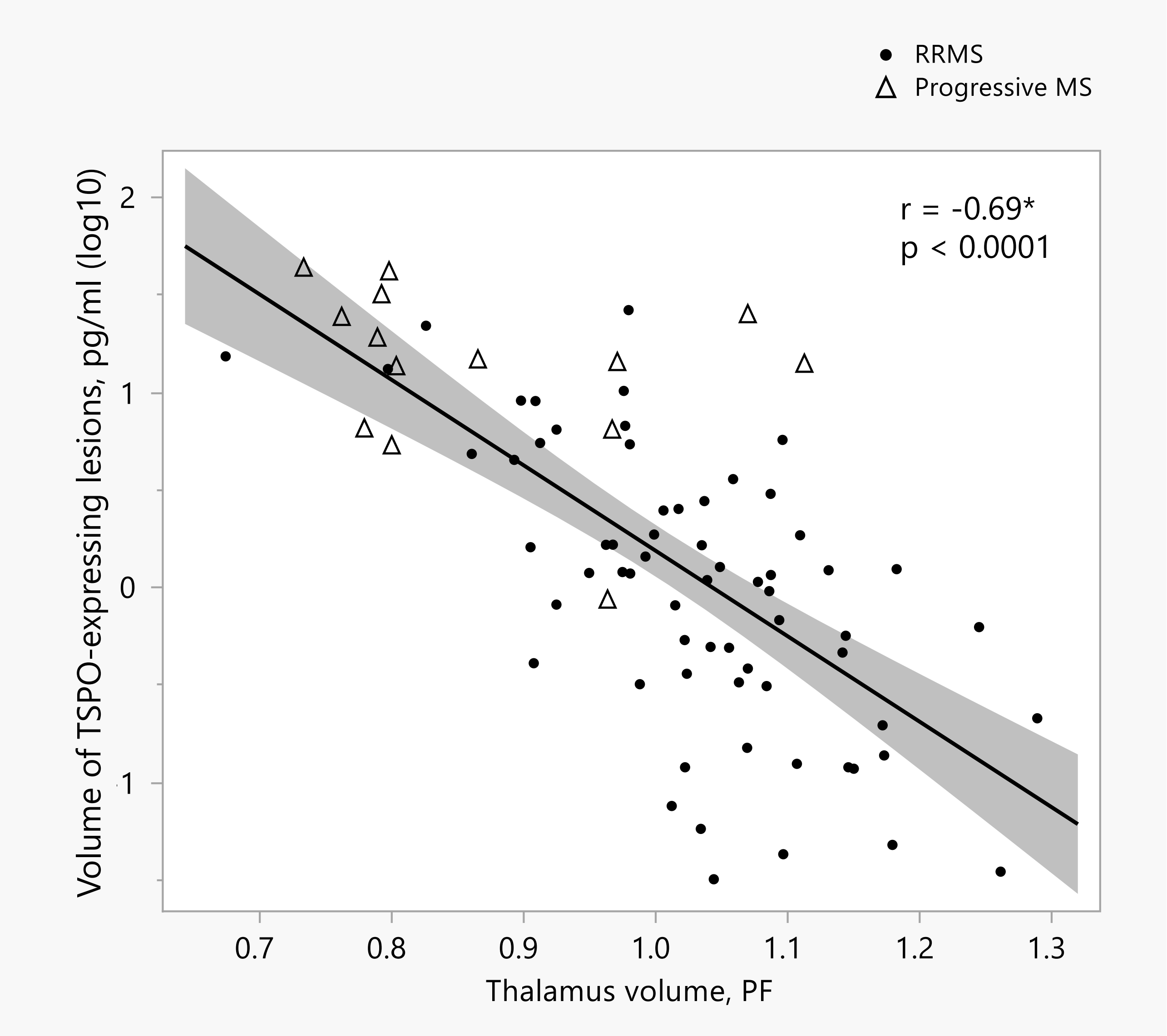

Additional Figure 1. Association between the volume of TSPO-expressing lesions (log10) and thalamus volume presented as a scatterplot with Pearson correlation coefficient. MS, multiple sclerosis; RRMS, relapsing remitting MS; TSPO, 18kDa translocator protein; PF, parenchymal fraction, %. * indicates a statistically significant association, the shaded area represents the 95% confidence interval.

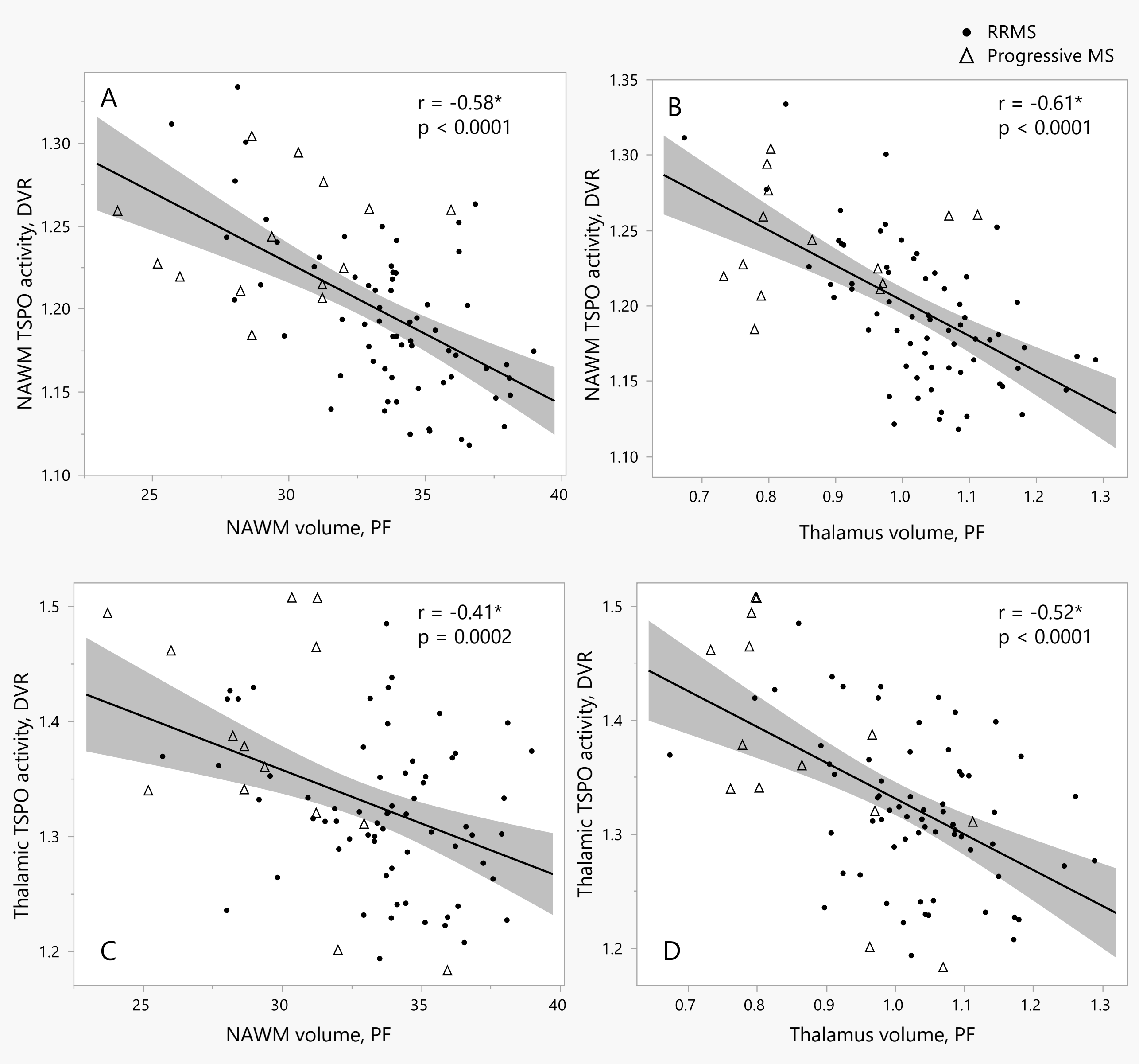

Additional Figure 2. Associations between TSPO activity in normal-appearing white matter (NAWM) (A, B) and thalamus (C, D) and the NAWM (A, C) and thalamus (B, D) volumes presented as scatterplots with Pearson correlation coefficients. MS, multiple sclerosis; RRMS, relapsing-remitting MS; TSPO, 18kDa translocator protein; DVR, distribution volume ratio; NAWM, normal-appearing white matter; PF, parenchymal fraction, %. * indicates a statistically significant association, the shaded area represents the 95 % confidence interval.

Additional Tables

Additional Table 1. The differences between men and women in basic clinical parameters and 18 kDa translocator protein positron emission tomography distribution volume ratios (DVRs) in the groups of people with MS and healthy control participants.

	People with MS			Healthy		
	men	women	*p*	men	women	*p*
n	25	55		3	8	
RRMS, n (%)*	19 (76)	47 (85)	0.35	na.	na.	
Age, years	46.6 (7.7)	45.6 (7.7)	0.61	44.7 (37.9, 46.0)	42.2 (35.3, 49.6)	0.78
sGFAP, pg/ml**	91.1 (58.8, 145.7)	92.1 (65.7, 120.5)	0.41	86.7 (33.5, 96.9)	55.5 (43.7, 78.9)	0.63
sNfL, pg/ml**	11.7 (8.3, 20.0)	8.5 (6.9, 13.5)	0.014	8.2 (3.1, 8.4)	8.5 (4.5, 10.0)	0.38
Brain TSPO activity, DVR	1.2 (0.02)	1.19 (0.03)	0.13	1.18 (1.17, 1.22)	1.18 (1.16, 1.19)	0.78
NAWM TSPO activity, DVR	1.22 (0.05)	1.19 (0.05)	0.011	1.2 (1.17, 1.25)	1.17 (1.12, 1.20)	0.28
Thalamic TSPO activity, DVR	1.37 (0.08)	1.31 (0.07)	0.0052	1.35 (1.33, 1.37)	1.31 (1.28, 1.33)	0.049

Unless otherwise stated, the results of people with MS are presented as mean (SD) and analysed with pooled t-tests; and the results of healthy control participants are presented as median (Q1, Q3) and analysed with Wilcoxon exact tests. RRMS, relapsing-remitting MS; GFAP, glial fibrillary acid protein; sNfL, serum neurofilament light; TSPO, 18 kDa translocator protein; DVR, distribution volume ratio; NAWM, normal-appearing white matter; na., not applicable. *Analysis conducted with Fisher's exact test. ** Presented as median (Q1, Q3) and analysis conducted with log10-transformed estimates.

Additional Table 2. The correlation coefficients between serum glial fibrillary acid protein (log10) and 18 kDa translocator protein binding in distinctive cortical and subcortical regions in the MS cohort (n=80).

	r/ρ	*p*
Frontal Cortex TSPO activity, DVR	-0.07	0.55
Parietal Cortex TSPO activity, DVR	-0.06	0.59
Temporal Cortex TSPO activity, DVR	0.09	0.41
Occipital Cortex TSPO activity, DVR	0.06	0.62
Cingulate cortex TSPO activity, DVR (log10)	0.03	0.77
Insular cortex TSPO activity, DVR	-0.17	0.14
Putamen TSPO activity, DVR	0.12	0.29
Caudate TSPO activity, DVR*	-0.10	0.40
Globus Pallidus TSPO activity, DVR	0.13	0.23
Hippocampus TSPO activity, DVR	-0.12	0.31

Unless otherwise stated, the results are presented as Pearson correlation coefficients and the level of statistical significance was set at p=0.01. TSPO, 18 kDa translocator protein; DVR, distribution volume ratio.
* Spearman’s rank correlation coefficient.

Additional Table 3. The correlation coefficients between serum glial fibrillary acid protein (log10) and the variables presented in Figures 2–4, in the groups of people with relapsing-remitting and progressive MS.

	RRMS, n=66		Progressive MS, n=14	
	r/ρ	*p*	r/ρ	*p*
Whole brain volume, PF	-0.18	0.15	-0.52	0.056
NAWM volume, PF	-0.16	0.19	-0.58	0.031
Cortical GM volume, PF	-0.16#	0.20	-0.48#	0.085
Thalamus volume, PF	-0.18	0.14	-0.63#	0.016
Volume of TSPO-expressing lesions, cm^3 (log10)	0.21	0.090	0.35#	0.23
Volume of TSPO-inactive lesions, cm^3 (log10)	0.15	0.23	0.34	0.24
T1 lesion load, cm^3 (log10)	0.22	0.079	0.37#	0.20
T2 lesion load, cm^3 (log10)	0.27	0.028	0.45	0.10
Brain TSPO activity, DVR	0.02	0.85	-0.13	0.66
NAWM TSPO activity, DVR	0.08	0.54	-0.10	0.74
Thalamic TSPO activity, DVR	0.20	0.10	0.25#	0.39
Perilesional TSPO activity, DVR	0.16	0.21	-0.10	0.75

Unless otherwise stated, the results are presented as Pearson correlation coefficients and the level of statistical significance was set at p=0.01. MS, multiple sclerosis; RRMS, relapsing-remitting MS; PF, parenchymal fraction; NAWM, normal-appearing white matter; TSPO, 18 kDa translocator protein; DVR, distribution volume ratio. #Spearman's rank correlation coefficient.

Additional Table 4. The results from a standard least squares regression model predicting serum glial fibrillary acid protein (log10) with age and the presence of disease modifying treatment as explanatory variables.

	β	VIF	DF	F Ratio	*p*	R^2
Age at sampling, years	0.29	1.00	1	7.1	0.0092	0.13
DMT [0]	0.20	1.00	1	3.4	0.069	

DMT [0], no disease modifying treatment; VIF, variance inflation factor; DF, degrees of freedom.

Additional Table 5. The results from additional standard least squares regression models predicting serum glial fibrillary acid protein (log10) with four explanatory variables.

	β	VIF	DF	F Ratio	*p*	R^2
T1 lesion load, cm^3	0.30	1.41	1	6.6	0.012	0.29
Thalamic TSPO activity, DVR	0.18	1.39	1	2.4	0.12	
Age at sampling, years	0.22	1.05	1	4.7	0.033	
DMT [0]	0.21	1.05	1	4.5	0.036	
T2 lesion load, cm^3	0.29	1.57	1	5.6	0.021	0.28
Thalamic TSPO activity, DVR	0.16	1.54	1	1.8	0.19	
Age at sampling, years	0.22	1.06	1	4.7	0.034	
DMT [0]	0.21	1.05	1	4.5	0.037	

TSPO, 18 kDa translocator protein; DVR, distribution volume ratio; DMT [0], no disease modifying treatment; VIF, variance inflation factor; DF, degrees of freedom.

Additional Table 6. The results from standard least squares regression models predicting serum glial fibrillary acid protein (log10) with thalamus volume, T2 lesion load/volume of TSPO-expressing lesions, age, and the presence of disease modifying treatment as explanatory variables.

	β	VIF	DF	F Ratio	*p*	R^2
Thalamus volume, PF	-0.17	1.93	1	1.5	0.22	0.28
T2 lesion load, cm^3	0.28	1.76	1	4.7	0.033	
Age at sampling, years	0.17	1.15	1	2.6	0.11	
DMT [0]	0.20	1.03	1	4.2	0.044	
Thalamus volume, PF	-0.17	1.79	1	1.8	0.19	0.29
Volume of TSPO-expressing lesions, cm^3	0.29	1.63	1	5.5	0.022	
Age at sampling, years	0.17	1.15	1	2.6	0.11	
DMT [0]	0.20	1.03	1	4.2	0.043	

PF, parenchymal fraction; DMT [0], no disease modifying treatment; TSPO, 18 kDa translocator protein; VIF, variance inflation factor; DF, degrees of freedom.

Additional Table 7. The results from a standard least squares regression model predicting thalamus volume (PF) with NAWM TSPO activity, thalamic TSPO activity, serum glial fibrillary acid protein, and age as explanatory variables.

	β	VIF	DF	F Ratio	*p*	R^2
NAWM TSPO activity, DVR	-0.40	1.39	1	18.9	< 0.0001	0.54
Thalamic TSPO activity, DVR	-0.27	1.37	1	8.7	0.0043	
sGFAP, pg/ml	-0.23	1.16	1	7.4	0.0083	
Age at sampling, years	-0.16	1.18	1	3.3	0.071	

NAWM, normal-appearing white matter; TSPO, 18 kDa translocator protein; DVR, distribution volume ratio; sGFAP, serum glial fibrillary acid protein; VIF, variance inflation factor; DF, degrees of freedom.

Additional Table 8. The results from standard least squares regression models predicting serum glial fibrillary acid protein (log10). Age and disease type (RRMS/progressive MS) were included as explanatory variables in all the models.

	β	VIF	DF	F Ratio	*p*	R^2
Whole brain volume, PF	-0.21	1.19	1	3.6	0.063	0.19
Age at sampling, years	0.14	1.26	1	1.4	0.24	
Disease type [RRMS]	-0.23	1.19	1	4.1	0.046	
Whole brain volume, PF	-0.22	1.20	1	3.9	0.051	0.21
Age at sampling, years	0.14	1.26	1	1.4	0.24	
Disease type [RRMS]	-0.19	1.25	1	2.8	0.10	
DMT [0]	0.16	1.05	1	2.4	0.12	
Cortical GM volume, PF	-0.29	1.38	1	6.0	0.016	0.21
Age at sampling, years	0.06	1.47	1	0.2	0.64	
Disease type [RRMS]	-0.24	1.17	1	4.6	0.035	
Cortical GM volume, PF	-0.31	1.39	1	6.9	0.010	0.24
Age at sampling, years	0.05	1.47	1	0.2	0.66	
Disease type [RRMS]	-0.20	1.22	1	3.1	0.083	
DMT [0]	0.18	1.06	1	2.9	0.093	

PF, parenchymal fraction; RRMS, relapsing-remitting multiple sclerosis; DMT [0], no disease modifying treatment; GM, grey matter; VIF, variance inflation factor; DF, degrees of freedom.
